# Genetic variations in sites of affinity between FVIII and LRP1 are not associated with high FVIII levels in venous thromboembolism

**DOI:** 10.1038/srep09246

**Published:** 2015-03-18

**Authors:** Luis F. Bittar, Lucia H. Siqueira, Fernanda A. Orsi, Erich V. De Paula, Joyce M. Annichino-Bizzacchi

**Affiliations:** 1 Hematology and Hemotherapy Center, University of Campinas, Campinas, SP, Brazil

## Abstract

Increased factor VIII (FVIII) levels are a prevalent and independent risk factor for venous thromboembolism (VTE). The low density lipoprotein receptor-related protein 1 (LRP1) has been associated with FVIII catabolism. After a median of 10 years of the first thrombotic episode, we evaluated FVIII activity levels in 75 patients with VTE and high FVIII levels and in 74 healthy controls. Subsequently, we evaluated the regions of *F8* and *LRP1* genes coding sites of affinity between these proteins, with the objective of determining genetic alterations associated with plasma FVIII levels. After a median time of 10 years after the VTE episode, FVIII levels were significantly higher in patients when compared to controls (158.6 IU/dL vs. 125.8 IU/dL; P ≤ 0.001]. Despite the fact that we found 14 genetic variations in *F8* and *LRP1* genes, no relationship was found between FVIII levels with these variations. We demonstrated a persistent increase of FVIII levels in patients with VTE, but in a much lower magnitude after 10 years when compared to 3-years after the episode. Moreover, we observed no relationship of genetic variations in the gene regions coding affinity sites between LRP1 and FVIII with FVIII levels.

Venous thromboembolism (VTE) is a multifactorial disease, and increased levels of coagulation factor VIII (FVIII) have been established as a prevalent and independent risk factor for the first episode and recurrent VTE^1^,^2^,^3^,^4^. We previously demonstrated that increased levels of FVIII are associated with VTE in Brazilian patients^5^. The main determinants of FVIII in plasma are von Willebrand factor (VWF) and ABO blood group^6^,^7^. Although FVIII is an acute phase protein, results from previous studies suggested that increased FVIII levels in VTE patients are independent of an acute reaction^8^,^9^,^10^,^11^ and a familial clustering was observed^12^,^13^,^14^. Analysis of FVIII gene showed several polymorphisms, but without any correlation with elevated FVIII levels^15^,^16^,^17^,^18^.

The low density lipoprotein receptor-related protein 1 (LRP1), also known as α2-macrogobulin receptor or CD91 is a large (600 kDa) multifunctional receptor which belongs to the low-density lipoprotein receptor family of endocytic receptors. LRP1 is involved in clearance of many different ligands, including FVIII^19^. The first evidence of the role of LRP1 in clearance of FVIII was simultaneously described by two studies. The authors showed by surface plasmon resonance analysis that FVIII binds to LRP1 with a moderate affinity. Moreover, assays in cell culture of fibroblasts and *in vivo* animal model showed that radioactive FVIII (^I-125^FVIII) specifically binds to LRP1 mediating the internalization and subsequent degradation of FVIII, indicating that LRP1 plays an important role in FVIII clearance^20^,^21^. Bovenschen et al^22^ confirmed the role of LRP1 in the clearance of FVIII employing an *in vivo* LRP1-deficient mouse model. Although several studies investigated the correlation between polymorphisms in LRP1 gene and plasma levels of FVIII and VTE^14^,^23^,^24^,^25^,^26^, only C663T polymorphism showed association with elevated FVIII levels and VTE risk^25^.

The ligand-binding sites within the extracellular domain of LRP1 are composed by four clusters of complement-type repeats (CRs), and each CR is formed by approximately 40 amino acids. Clusters II and IV were shown to be responsible for the binding of majority of LRP1 ligands, including FVIII. The LRP1 cluster II consists of 8 CRs, and it was demonstrated that FVIII has affinity for the first six CRs of cluster II^27^,^28^,^29^. Within the FVIII molecule, two high-affinity binding sites for LRP1 have been identified: the region 484–509 of the A2 domain and the region 1811–1818 of the A3 domain; and a low-affinity site in the region 2303–2333 of the C2 domain^30^,^31^,^32^,^33^.

Therefore, genetic variations in these regions could potentially affect FVIII clearance, thereby influencing the plasma FVIII levels. Herein, we investigated the presence of genetic variations in gene regions coding sites of affinity between FVIII and LRP1 in patients with history of VTE and persistently elevated FVIII levels.

## Results

Median age of the 75 patients (22 male e 53 female) was 47 years, and median time after the fist VTE episode was 10 years (range 8–15). The control group consisted of 74 participants (28 male and 46 female) with a median age of 45 years. There was no significant difference between patients and controls related to gender, age, and ABO blood group (Table 1).

Venous thromboembolism was spontaneous in 28 (37.3%) patients. In the remaining 47 (62.7%) patients, associated risk factors were surgery (8.0%), immobilization (17.3%), oral contraceptive use (21.3%), pregnancy and puerperium (12.0%), among other less prevalent causes (4.1%). The sites of VTE were: lower limbs (88,1%), splenic-portal circulation (7,4%) and upper extremities (4,5%). Four (5,3%) patients with venous thrombosis of the lower limbs also presented pulmonary embolism.

Table 1 shows that in the first assessment, with a median time of the first VTE episode of 3 years (range 1–8), FVIII levels was 232.4 IU/dL, and significantly higher than in controls (126.9 IU/dL; P ≤ 0.001). In the second assessment, with a median time of the first VTE episode of 10 years (range 8–15), FVIII levels was still significantly higher in patients (158.6 IU/dL) than in controls (125.8 IU/dL; P ≤ 0.001), although with a lower magnitude.

In the first assessment, all 75 patients included in this study had FVIII above the P90 (200.0 IU/dL) since thus was a prior inclusion criteria for this study. During the second analysis, the P90 was 170.3 IU/dL, and only 26 patients (34.7%) remained above this percentile. Therefore, we observed a significant decrease in the number of patients with increased FVIII levels in the second analysis (P ≤ 0.001). CRP levels in patients with VTE were between normal values (0.11 mg/dL) but significantly lower when compared to the first measure with 3-year median after venous thromboembolism episode (0.21 mg/dL; P = 0.003).

We identified 14 genetic variations, including 12 in *LRP1* gene and 2 in *F8* gene. Molecular alterations previously described were classified as mutation (allelic prevalence < 1%) or polymorphism (allelic prevalence ≥ 1%) according to their prevalence in the population of NCBI database, composed of 1000 healthy individuals (2000 alleles). Novel genetic variations were classified according to their prevalence in the control group of the study, thus, two were classified as polymorphisms and twelve as mutations. Of the 12 mutations identified, three had not yet been described. Despite the identification of several genetic alterations, no significant difference was observed in their prevalence between patients and controls, with no association with VTE history (Table 2).

The comparison of plasma FVIII levels according to genotypes of LRP1 polymorphisms in the VTE patients group did not show statistical differences (Table 3).

## Discussion

The mechanisms underlying the elevation of FVIII levels in VTE patients are not totally established. The main determinants of FVIII in plasma are the von Willebrand factor (VWF) level and the ABO blood group. Moreover, age and ethnicity also have an important role in the regulation of FVIII levels. Immediately after its release into the circulation, FVIII is caught into a tight non-covalent complex with the VWF. The VWF acts as a natural carrier of FVIII, and the complex VWF/FVIII is crucial for the survival of FVIII in the blood circulation by protects it against impropriated proteolytic activation and premature clearance. Therefore, it is not surprising that plasma VWF level plays a critical role in regulating plasma FVIII levels. However, the mechanisms underlying the inter-individual variability of plasma FVIII levels are not yet fully understood, and these main determinants explain only a part of FVIII levels variation. Thus, it has been suggested that others unknown important factors (mainly genetic factors) could have participation on FVIII variation^34^.

Previous studies that evaluated patients with VTE and increased FVIII levels suggested a familial clustering of high FVIII levels^12^,^13^,^14^. For example, in a retrospective study of 177 VTE patients with high FVIII levels, Bank *et al.* (2005) observed that 40% of their first-degree relatives also presented FVIII levels above the 75th percentile of the normal population. Moreover, these first-degree relatives with elevated plasma FVIII levels also demonstrated increased risk for VTE^13^.

In the present study, we hypothesized that genetic variations in gene regions coding the affinity sites between FVIII and LRP1 might influence plasma FVIII levels, and that a cohort of VTE patients with persistently elevated FVIII levels might be an interesting target for screening these genetic variations. By selecting this population, we hoped increase the possibility for detecting clinically relevant mutations or polymorphisms associated with elevated FVIII levels. With this strategy, we identified two mutations in the analyzed regions of *F8* gene, one in intron 9 and other in the 3′ UTR region, present in one VTE patient. Morange *et al.* (2005) evaluated these same regions of *F8* gene, in 10 healthy individuals with the top higher and lower FVIII levels of 424 healthy individuals. No polymorphism was detected in these regions of the *F8* gene^14^. In accordance with our results, genetic variations in these *F8* gene regions are probably not associated with elevated FVIII levels.

Two studies simultaneously identified the LRP1 as a first candidate clearance receptor for FVIII^20^,^21^, and in the last decade, several studies have analyzed the influence of LRP1 on FVIII levels in humans^14^,^25^,^26^,^27^,^28^. In particular, investigators have focused their research on analyze the association between LRP1 polymorphisms and plasma FVIII levels, and VTE risk. Association between LRP1 D2080N and –25C/G polymorphisms with decreased FVIII levels^14^,^26^, and LRP1 C663T polymorphism with increased FVIII levels and with a three-fold increased risk of VTE have been described^25^.

Although we identified 12 mutations/polymorphisms in the region of *LRP1* gene, these were not associated with FVIII levels. Our results suggest no association of genetic variations located in the first six CRs of cluster II of LRP1 with plasma FVIII levels, but we could not ruled out the role of variations affecting other important affinity region, localized in cluster IV^27^,^28^,^29^. We can also consider that despite the efficiency which LRP1 binds FVIII and mediates its intracellular degradation, the absence of LRP1 resulted only in a partial inhibition of FVIII degradation when evaluated *in vivo* assays, indicating that other LRP1-independent pathways are involved in FVIII uptake^22^,^35^.

Finally, in this study, we showed that patients with a median of 10 years after the first episode of VTE presented higher FVIII levels when compared to healthy controls, but the magnitude of this difference was significantly lower than that observed at an earlier time point. These findings suggest the presence of stimulus to increase FVIII levels that could be stronger during the initial years after the VTE episode. CRP is expressed at low levels in the absence of an acute inflammatory stimulus, and the results found in our patients suggested that, they did not present an acute inflammatory response in both assessments. Our results corroborate other studies that analyzed inflammatory markers after VTE and almost all demonstrated decline of CRP after a short time^11^,^36^,^37^ Thus, these findings suggested that increased FVIII levels observed in patients with previous VTE be, in part, consequence of a sub-clinical chronic inflammation, not revealed by CRP. In accordance with this hypothesis, Bouman *et al.* (2014) performed an evaluation 63 months after the acute thrombotic episode describing increased levels of IL-6 in patients when compared to controls^38^. Thus, we cannot rule out that even years after the thrombotic episode, a sub-clinical chronic inflammatory state could contribute to high levels of FVIII in VTE patients.

Thus far, strengths of the present study are the evaluation of specific gene regions coding the affinity sites between FVIII and LRP1 that might influence plasma FVIII levels, in a well-defined population of cases with a history of high FVIII levels and matched controls. However, an important limitation of this study is the relatively small sample size, which should be considered in the evaluation of associations between the genetic alterations and high FVIII levels. Therefore, this was an exploratory study and a relationship between the genetic variations found, high FVIII levels and VTE remains to be clarified by larger studies.

## Conclusions

We demonstrated a persistent increase of plasma FVIII levels in a subset of patients with VTE, but in a much lower magnitude after 10 years of VTE episode when compared to the first evaluation 3-years after the episode. Moreover, we observed no relationship between genetic variations in the gene regions coding sites of affinity between LRP1 and FVIII proteins and FVIII levels.

## Methods

### Study Population

Written informed consent was obtained from all participants, and the study was approved by the Ethics Committee of University of Campinas. All the methods were carried out in accordance with the approved guidelines. This was a case-control study and initial cohort consisted of 314 adult patients with a first episode of VTE, between January 1990 and September 2004, followed up at the Hemostasis and Thrombosis Clinic, at University of Campinas, Brazil. Patients with cancer, liver, renal, or systemic inflammatory diseases were excluded. VTE was confirmed by imaging tests. In a first assessment, with a median time of three years after the acute episode, FVIII levels were evaluated in all 314 patients and in 314 matched controls, without a medical history of VTE from the same geographic region and ethnic background of the patients. Seven years later, all 104 patients from this initial cohort who originally presented FVIII levels above the 90^th^ percentile (P90) were recruited for a second assessment, but only 75 patients (72.1%) agreed to participate. Seventy-four healthy controls were again selected according to age, gender, ABO blood group, geographic region, ethnic background, and the same exclusion criteria were used for the study entry.

### Laboratory Methods

After an overnight fast, venous blood samples (18 mL) were collected from all participants, from the antecubital vein into into Vacuette® tubes (Greiner Bio-One, Austria): 0.129 mmol/L trisodium citrate tube, ethylenediaminetetraacetic (EDTA) tube, and Z Serum Sep Clot Activator tube. Samples were immediately centrifuged for 20 minutes at 1500 *g*, and plasma/serum were immediately frozen and stored at −80°C.

### FVIII Activity

FVIII activity levels were measured by a one-stage clotting assay with FVIII-deficient plasma (Siemens, Marburg, Germany) as recommended by the manufacturer. FVIII were determined in duplicate on automated coagulation analyzer (BCS XP, Siemens, Marburg, Germany). This methodology was performed in both assessments. Normal range was 62 to 151 IU/dL.

### C-reactive protein

Serum high sensitive C-Reactive protein (hs-CRP) levels were determined by a nephelometric method (*Siemens, Marburg, Germany*), on Siemens BN ProSpec analyzer. Normal range was < 0.50 mg/dL.

### ABO blood group

ABO blood group was determined by agglutination and adsorption-elution test.

### Sequencing of the LRP1 and F8 gene regions potentially involved in LRP1-FVIII binding

Three regions were selected in *F8* gene: (i) exons 10 and 11 (containing the site Arg484-Phe509 in the A2 domain), (ii) exon 16 (containing the site Lys1804-Ala1834 in the A3 domain), and (iii) exon 26 (containing the site Thr2303-Tyr2333 in the C2 region). In the *LRP1* gene, exons 14 to 20 (containing the site of the first six CRs of cluster II) were selected. All these regions were amplified by polymerase chain reaction (PCR). Primer sequences, PCR products and length of PCR products are listed in the Supplementary Table S1.

PCR products were checked on 2% agarose gels (Uniscience, Brazil) stained with 2 ug/ml ethidium bromide (Invitrogen, USA), and purified by commercial kit GFX PCR DNA and Gel Band Purification Kit (GE Healthcare). Subsequently, these products were sequenced by the Big Dye Terminator Cycle Sequencing Ready Reaction Kit (Life Technologies, USA) according to manufacturer's instructions for subsequent electrophoresis in automatic sequencer ABI PRISM® 3500 Genetic Analyzer (Applied Biosystems, USA). The analysis of sequencing was performed by the Chromas software (version 2.3.0), comparing the samples sequences to database of The National Center for Biotechnology Information (NCBI), NG_016444.1 for *LRP1* gene and NG_011403.1 for *F8* gene.

### Statistical analysis

Continuous variables were described as median and interquartile range. Medians between patients and controls were compared by the Mann-Whitney test, and categorical variables were compared by the Fisher's exact test. Genotype frequencies between patients and controls were compared by the Fisher's exact test. Medians of FVIII levels according the genotypes were compared by Mann-Whitney or Kruskal-Wallis tests. A P value <0.05 was considered statistically significant and all tests were two-tailed. All analysis was performed by the R Foundation for Statistical Computing, version 3.0.1.

## Author Contributions

L.F.B., L.H.S., F.L.O., E.V.P. and J.M.A-B. designed the experiments and analyzed the data. L.F.B., E.V.P. and J.M.A.-B. wrote the manuscript text. L.F.B. performed the experiments and L.H.S. assisted with primers design. All authors reviewed the manuscript.

## Supplementary Material

Supplementary Information Supplementary Table 1

## Figures and Tables

**Table 1:** Baseline Characteristics of Patients with Venous Thromboembolism and Control Group

	VTE Patients (N = 75)	Controls (N = 74)	P*
Age - Median (*Interquartile Range*)	47 (40–55)	45 (36–51)	0.28
Gender - Female/Male (%)	70.7%/29.3%	62.2%/37.8%	0.29
ABO Blood Group - Non-O/O (%)	68.0%/32.0%	67.6%/32.4%	1.00
FVIII - First Assessment (2004) [IU/dL] *(Interquartile)*	232.4 (216.2–286.1)	126.9 (98.8–145.8)	≤ **0.001**
FVIII - Second Assessment (2011) [IU/dL] *(Interquartile)*	158.6 (148.5–171.0)	125.8 (93.4–148.6)	≤ **0.001**

This table shows baseline characteristics of VTE patients and controls.

*P values were calculated by the Fisher's exact test for categorical variables and Mann-Whitney test for continuous variables.

Abbreviations: FVIII = factor VIII; VTE = venous thromboembolism.

**Table 2:** Genetic variations identified in *LRP1* and *F8* genes

Nucleotide change	Amino acid change	Gene Region	NCBI Code	Prevalence of mutant allele in the NCBI (%)*	Classification	Prevalence of mutant allele in the study Patients/Controls % (N)	P*
C 2767 T	Arg 767 Arg	*LRP1* Exon 14	rs145618022	0.2	Mutation	T: 0.67 (1)/0.68 (1)	1
C 2805 T	Thr 780 Ile	*LRP1* Exon 14	rs34492744	0.3	Mutation	T: ZERO (0)/0.68 (1)	1
C 2870+167 T	NA	*LRP1* Intron 14	rs35306126	0.6	Mutation	T: ZERO (0)/0.68 (1)	1
C 2870+197 T	NA	*LRP1* Intron 14	rs34709055	0.6	Mutation	T: ZERO (0)/0.68 (1)	1
G 2996+26 A	NA	*LRP1* Intron 15	rs138196021	0.1	Mutation	A: 0.67 (1)/ZERO (0)	1
C 3137+54 T	NA	*LRP1* Intron 16	rs181881519	0.1	Mutation	T: 0.67 (1)/ZERO (0)	1
G 3137+81 A	NA	*LRP1* Intron 16	rs1800174	40.0	Polymorphism	A: 39.3 (59)/35.8 (53)	0.59
G 3137+111 A	NA	*LRP1* Intron 16	Novel	NA	Mutation	A: 0.67 (1)/ZERO (0)	1
G 3264 -172 A	NA	*LRP1* Intron 17	Novel	NA	Mutation	A: ZERO (0)/0.68 (1)	1
C 3264 - 3 T	NA	*LRP1* Intron 17	Novel	NA	Mutation	T: 0.67 (1)/ZERO (0)	1
G 3376 A	Ser 970 Ser	*LRP1* Exon 18	rs78054559	0.1	Mutation	A: 0.67 (1)/ZERO (0)	1
T 3380+114 C	NA	*LRP1* Intron 18	rs34474417	2.2	Polymorphism	C: 2.66 (4)/ZERO (0)	0.13
T 1444 - 22 C	NA	*F8* Intron 9	rs5986899	0.7	Mutation	C: 0.67 (1)/ZERO (0)	1
C 7053 + 32 T	NA	*F8* 3′ UTR	rs5986887	0.9	Mutation	T: 0.67 (1)/ZERO (0)	1

This table shows information about mutations and polymorphisms found in the study, including their prevalence in patients and controls, and NCBI database.

*Genetic alterations previously described were classified as mutation (allelic prevalence < 1%) or polymorphism (allelic prevalence ≥ 1%) according to their prevalence in the population of NCBI analysis, composed of 1000 healthy individuals (2000 alleles).

**P values were calculated by the Fisher's exact test. Abbreviations: NCBI = The National Center for Biotechnology Information; Arg = Arginine; Thr = threonine; Ile = Isoleucine; Ser = Serine; 3′ UTR = 3′ untranslated region; NA = Not Applicable.

**Table 3:** Plasma FVIII levels according to genotypes in VTE patients

Polymorphisms	N	FVIII - 1° Assessment [IU/dL]*	FVIII - 2° Assessment [IU/dL]*
G 3137+81A			
GG	27	238.3 (219.3–276.3)	164.0 (153.3–174.5)
GA	37	252.6 (223.6–326.0)	156.0 (150.5–172.8)
AA	11	240.8 (223.5–283.0)	158.0 (134.0–160.2)
		*P = * 0.66**	*P = * 0.47**
T 3380+114 C			
TT	71	239.9 (218.3–298.1)	157.1 (148.0–171.0)
TC	4	233.4 (212.7–241.7)	168.0 (163.0–173.0)
		*P = * 0.43**	*P = * 0.33**

This table shows plasma FVIII levels in VTE patients according their genotypes for LRP1 polymorphisms.

*Results are expressed as median (interquartile range).

**P values were calculated using the Kruskal-Wallis test for G 3137+81A polymorphism, and Mann-Whitney test for T 3380+114 C polymorphism.
