# Supplementary material for: Genetic variations in sites of affinity between FVIII and LRP1 are not associated with high FVIII levels in venous thromboembolism
Source: Sci Rep. 2015 Mar 18;5:9246. doi: 10.1038/srep09246 (PMC4363825; doi:10.1038/srep09246)
Supplement: Supplementary Information — Supplementary Table 1 [file srep09246-s1.pdf]

Genetic variations in sites of affinity between FVIII and LRP1 are not associated with high FVIII levels in venous thromboembolism

Luis Fernando Bittar, Lucia Helena Siqueira, Fernanda L. Orsi, Erich Vinícius De Paula & Joyce M. Annichino-Bizzacchi.

Supplementary Table 1: Regions of *LRP1* and *F8* genes, product length, and primer sequences:

**Region**	**Gene**	**Product Length**	**Primer Sequences**
LRP1 Ex.14/15	*LRP1*	891 pb	Fd: 5' TGGGGCTCCCAGGCTAATGGGGG 3'<br>Rev: 5' TGGGACCCTGGATCCTCCCCTCCC 3'
LRP1 Ex. 16/17	*LRP1*	527 pb	Fd: 5' CTTGTCCCACGACCGGGGTCTGACT 3'<br>Rev: 5' GGCCCCCTCGTCTCCCCTGCCTC 3'
LRP1 Ex. 18/19	*LRP1*	999 pb	Fd: 5' GGGGATGGAGCCCTGAGCAGGAGTG 3'<br>Rev: 5' GCCTTCCCTGGAGGGCCTGGCTG 3'
LRP1 Ex. 20	*LRP1*	295 pb	Fd: 5' GGCAGGGGGCCCAAGCTGGGATC 3'<br>Rev: 5' GGGGGCTCGATCTCATCCCCACCC 3'
F8 Ex. 10	*F8*	345 pb	Fd: 5’ AGCCTCAAATTACTATAATG 3’<br>Rev: 5’ ACTTTAGACTGGAGCTTGAG 3’
F8 Ex. 11	*F8*	362 pb	Fd: 5’ CCCTTGCAACAACAACATGA 3’<br>Rev: 5’ TTTCTTCAGGTTATAAGGGGACA 3’
F8 Ex. 16	*F8*	456 pb	Fd: 5’ AGCATCCATCTTCTGTACCA 3’<br>Rev: 5’ TTGCACGTAGGATAAATATC 3’
F8 Ex. 26	*F8*	344 pb	Fd: 5’ ACTGGAAACAACTAGAAGTG 3’<br>Rev: 5’ TTAGCACAAAGGTAGAAGGC 3’

This table shows the analyzed regions of *LRP1* and *F8* genes, PCR product lengths, and primer sequences. *Abbreviations*: Ex = exon; Fd = forward primer; Rev = reverse primer; pb = pairs base.
